# Immune Responses to Methicillin-Resistant *Staphylococcus aureus* Infections and Advances in the Development of Vaccines and Immunotherapies

**DOI:** 10.3390/vaccines12101106

**Published:** 2024-09-27

**Authors:** John Scully, Abu Salim Mustafa, Asma Hanif, Javed H. Tunio, Shumaila Nida Javed Tunio

**Affiliations:** 1 Department of Biomedical Sciences, University of Pikeville, Pikeville, KY 41501, USA; johnscully@upike.edu; 2 Department of Microbiology, College of Medicine, Kuwait University, Jabriya 46300, Kuwait; abu.mustafa@ku.edu.kw; 3 Department of Restorative Sciences, College of Dentistry, Kuwait University, Kuwait City 12037, Kuwait; asmaa.haneef@ku.edu.kw; 4 Department of Internal Medicine, Carver College of Medicine, University of Iowa, Iowa City, IA 52242, USA; jtunio@iowa.edu

**Keywords:** MRSA, immune responses, vaccines, immunotherapies

## Abstract

*Staphylococcus aureus* (SA) is a major bacterial pathogen and causes a wide range of clinical infections in humans leading to severe outcomes including meningitis, endocarditis, and sepsis. This literature review examines studies on host immune responses after infections with SA and methicillin-resistant *Staphylococcus aureus* (MRSA) and their immune evasion mechanisms. Furthermore, information about vaccines and immunotherapies against SA and MRSA is reviewed. We found promising toxoid vaccine approaches, which deserve further research. We also found support for antitoxin therapies and immunomodulating therapies as high-potential research areas. Although many promising vaccines and immunotherapy candidates have been studied in animal models, more human clinical studies are needed to confirm their long-term safety and efficacy.

## 1. Introduction

*Staphylococcus aureus* (SA) is a Gram-positive, non-motile bacterium commonly found in the human environment. A significant portion (up to 30%) of the population is colonized with SA, which resides primarily in the gastrointestinal tract, nares, and skin [1]. SA causes many complex infections that include skin and soft tissue infections, bone and joint infections, meningitis, osteomyelitis, pneumonia, and infective endocarditis [2]. The high-risk groups for *SA* infection include people receiving immunosuppressive and anti-cancer therapies, young children, and infants with a low birth weight [3].

Methicillin-resistant *Staphylococcus aureus* (MRSA), a prominent human health threat, can resist treatment by many antibiotics including methicillin, penicillin, oxacillin, cloxacillin, cefazolin, and cefoxitin [4]. Frequent use of antibiotics in the clinical setting has contributed significantly to the development and spread of methicillin- and multidrug-resistant SA [1]. Initially, the organism spread primarily in the clinical setting, but increasing virulence and antibiotic resistance has increased the spread and transmission of community-associated MRSA and the colonization of healthy individuals with methicillin-resistant variants [1].

Human immune responses to SA are diverse, and many factors may play a role in the susceptibility to infection and the outcome of infection. Different responses from various cell types including neutrophils, macrophages, T lymphocytes, and B lymphocytes are associated with positive or negative outcomes in both humans and animal models [5]. Furthermore, SA variants with a high pathogenicity release a variety of antigens and toxin molecules to avoid immunodetection and promote immune tolerance [5].

Many researchers have developed vaccine candidates to prevent SA and MRSA infection. Although many of these vaccine candidates have shown promising results in animal models, no SA or MRSA vaccine has been approved by the FDA for use in humans [5]. Various approaches have been used in the published literature to develop potential vaccines, including opsonization and toxoid vaccines [5]. Many of these approaches also utilize various adjuvants and generate impressive immune responses in animal models.

The increasing burden of MRSA and the increasing risk for the epidemic spread of MRSA, merits the investigation of alternative therapies to combat MRSA. The suggested new therapies include novel antibiotics, therapeutic vaccines, and immunotherapies. Potential immunotherapies include monoclonal antibodies, enzyme inhibitors, and genetic therapies. Monoclonal antibody approaches have diverse targets, including staphylococcal superantigens, toxins, lysins, and lipoteichoic acids. In the absence of a vaccine and the decreasing number of effective antibiotics, research to develop alternative therapies could lead to significant breakthroughs.

### 1.1. Immune Responses to SA and MRSA

After infection with SA and MRSA, the activated immune response is quite complex (Table 1). An important research topic in the context of vaccines and immunotherapeutics for SA and MRSA is the humoral, cell-mediated, and innate immune responses associated with infection and positive or negative outcomes. For example, recurrent infections with SA provide an important patient group to study, as differences in their immune system may contribute to their susceptibility to SA infections. A prospective study in children with recurrent SA infections found that the children susceptible to recurrent infections had significantly impaired effector T cell function [6]. This suggests that cell-mediated immunity may play a significant role in SA immunity. Additionally, based on murine models, it is likely that decreased IL-1β may significantly impair adaptive immune responses and promote increased inflammation in patients with MRSA pneumonia [7]. Follow-up studies are needed to determine whether decreased IL-1β levels are also associated with increased inflammation in MRSA pneumonia in humans.

T cell-mediated immunity, especially the Th1 and Th17 immune responses, appears to be one of the most critical elements in successfully controlling SA infections [8]. Some examples of the importance of the Th17 response are the predisposition toward recurrent SA infections among those with STAT3 loss-of-function mutations and hyper IgE syndrome due to impaired Th17 response [8]. Additionally, SA avoids humoral immune responses through SA protein A antagonism of the Fc–BCR interaction and inhibition of opsonophagocytosis mediated through the blockage of Fc [8]. Additionally, a high frequency of anti-SA antibodies is expected from early childhood into adulthood. However, anti-SA antibodies are poorly effective against SA, suggesting that SA may be able to antagonize host humoral immunity mechanisms [8].

Gamma–delta and alpha–beta T cells also play an important role in controlling SA infection [9]. Gamma–delta T cell-knockout mice experience more severe skin disease upon SA challenge than wild-type mice [9]. Transfer of gamma–delta T cells from mice previously infected with SA provides protection against SA in naïve mice [9]. Gamma–delta T cells may provide protection against SA through a strong induction of the Th17 immune response [9]. Alpha–beta T cells make up the majority of the T cells and produce CD4 or CD8 markers on the cell surface [9]. CD4 cells differentiate into Th-1, Th2, and Th17 cells in addition to other differentiations, and they induce a protective neutrophil-mediated response via the Th17 differentiation and secretion of Th17 cytokines [9].

A subclass of human T cells, group 1 CD1-restricted T cells, recognize lipid antigens and may play a critical role in controlling systemic SA [10]. Transgenic mice expressing human CD1 molecules showed significantly increased CD1 expression ten days after infection and significantly reduced kidney pathology compared with wild-type mice [10]. Another study showed that mice expressing type II NK T cells recognizing the lipid-presenting CD1d variant of MHC I had sufficient protection against systemic MRSA infection through IFN-gamma secretion [11]. It was also found that type II NK T cell-mediated IFN-gamma secretion was most strongly activated by exposure to phosphatidylglycerol, lysyl-phosphatidylglycerol, and polar lipids from the SA membrane [11].

Although mice studies are cheap, relatively easy to perform, and provide insights into immune responses, further studies of human immunity are necessary to determine what role different T cell subtypes and lipid antigen recognition have in human SA immunity. Ideally, studies performed in vitro, genomic analysis in afflicted patients, and other non-invasive studies of humans wherein ethically and logistically feasible should be performed to assess what aspects of murine SA immunity are applicable in human infections.

In addition to the activity of T cell-mediated immunity in protecting against MRSA infection, innate immunity against the SA bacterium is a critical component to preventing severe SA infections [12]. The skin is an essential barrier against the hematogenous spread of MRSA and SA infections. In SA skin infections, innate immune activity is stimulated by increased IL-6 on day 2, IL-17 on day 7, and a strong Th17 response in the skin [12]. Innate immunity against SA is most likely mediated through the recognition of SA pathogen-associated molecular patterns such as lipoteichoic acid, peptidoglycan, glycopolymers, RNA, and unmethylated CpG DNA by Toll-like receptors [9].

Dendritic cells are also highly important in performing immunological synapses and triggering a balanced Th1/Th17 response against SA infection [13]. Dendritic cells exposed to live SA triggered the balanced Th1/Th17 response via IL-12, TNF-alpha, and IL-6, while DCs exposed to heat-inactivated SA produced 6 to 10 times lower cytokines [13]. This difference in reactivity is essential in the context of inactivated vaccines. Inducing tolerogenic immune phenotypes is also a critical part of pathogenesis for SA. Community-associated MRSA variants (CA-MRSAs) often depend on the secretion of phenol-soluble modulins to modify the host immune response [14]. Phenol-soluble modulins in CA-MRSA infections induce a tolerogenic DC phenotype, and this leads to the priming of CD4+ CD25+ FoxP3+ Tregs and impairment of the cell-mediated immune response against CA-MRSA [14]. Additionally, models have suggested that DC may be killed by SA alpha-toxin, and this may further prevent the activation of protective immune memory [9].

**Table 1:** Immune responses to SA and MRSA infections.

Immune Response	Relevance	Reference
Effector T cell response	Critical to effective control of SA infection, frequently impaired in children with recurrent infections	[6]
Th1–Th17 balance	Critical to effective cell-mediated immunity against SA	[8]
Th2 response	Generally ineffective due to protein A blockage of opsonophagocytosis	[8]
CD1-T cell response	Recognition of SA membrane lipid antigens contributes to SA immune response	[10,11]
Dendritic cells	Performance of immunologic synapse, triggering of balanced Th1–Th17 response	[13]
IgE	May be produced in response to SA extracellular vesicles and may contribute to disease through a hypersensitivity reaction	[15]

### 1.2. Pathogenic and Immune Evasion Mechanisms Used by SA and MRSA

MRSA and SA produce several toxins involved in antagonizing the host immune system and mediating disease (Table 2). Toxic shock syndrome toxin 1 (TSST-1) is a superantigen produced by SA that binds MHC II and T cell receptors and activates up to 20% of the peripheral T cells [8]. TSST-1 mediates shock via extremely high levels of interleukin 1 and TNF-alpha secretion causing high fever, increased capillary permeability, and systemic hypotension [16]. TSST-1 is reported to cause 100% of vaginal tampon associated toxic shock syndrome cases and 50% of non-menstrual toxic shock syndrome cases [16]. Staphylococcal enterotoxin B is another superantigen that acts via the same mechanism as TSST-1 and has a remarkable ability to resist both heat and acid [8,17].

Staphylococcal pore-forming toxins have cell killing effects via the disruption of the cell membrane [17]. Staphylococcal alpha-hemolysin is a protein with pore-forming and proinflammatory activity that demonstrates killing activity against host erythrocytes, monocytes, macrophages, lymphocytes, fibroblasts, and endothelial cells [17]. Staphylococcal Panton–Valentine leukocidin, is another pore-forming toxin produced by SA [17]. Panton–Valentine leukocidin exhibits killing ability against polymorphonuclear cells, monocytes, and macrophages [17]. Additionally, Panton–Valentine leucocidin is a major virulence factor for staphylococcal necrotizing pneumonia and skin and soft tissue infections.

SA also produces several exfoliative toxins: exfoliatin A, exfoliatin B, and exfoliatin D [17]. These toxins are capable of cleaving desmosomal cadherin desmoglein1 (Dsg1) [17]. Exfoliatin toxins mediate staphylococcal skin infections such staphylococcal scalded skin syndrome [17]. Although rarely fatal in children, staphylococcal skin infections have a much higher mortality rate in adults despite antibiotic therapy [17]. Exfoliatin toxins also demonstrate superantigen activity and can spread into the bloodstream causing shock [17].

Several virulence factors provide SA with the ability to invade and attach to host tissues. Clumping factors A and B (ClfA and ClfB) play a critical role in attachment to and colonization of valvular tissue in SA-mediated bacterial endocarditis [2]. Fibronectin-binding protein A and B facilitate binding to fibronectin and fibrinogen and play a role in endothelial cell invasion and inflammation [2]. Clf, FnBP, and serine aspartate repeat protein (SDrE) induce platelet aggregation and may play a role in disseminated intravascular coagulation events in SA bacteremia [2].

MRSA may also antagonize the immune system via extracellular vesicle release [15]. Mice immunized with MRSA extracellular vesicles from clinical isolates died within one day of the MRSA challenge [15]. Immunization with MRSA extracellular vesicles demonstrated massive proinflammatory cytokine and IgE responses [15]. This suggests that some of the systemic effects of MRSA may be mediated through an IgE-mediated hypersensitivity reaction [15].

The presence of polysaccharide capsules in MRSA and SA clinical isolates may also be an important virulence factor in invasive staphylococcal infections caused by encapsulated strains. Approximately 90% of clinically isolated SA infections include capsules [17]. SA polysaccharide capsules consist of high-molecular-weight N-acetyl-D-fucosamine, N-acetyl-L-fucosamine, and N-acetyl-D-mannosaminuronic acid [17]. These capsules may, along with SA protein A, work to inhibit opsonophagocytosis, allowing for an invasive SA infection [17]. Although many SA strains isolated clinically have capsules, the USA300 MRSA strain does not show capsule formation [18].

**Table 2:** MRSA and SA pathogenic mechanisms.

Pathogenic Toxins	Consequences	References
Protein A	Inhibition of opsonophagocytosis via binding the Fc region of immunoglobulins	[8]
Phenol-soluble modulins	Induce tolerogenic DC phenotype, leads to priming of Foxp3+ Tregs	[14]
TSST-1	Binds MHC-II and TCRs, activates high levels of T cells leading to shock	[8,16]
Staphylococcal enterotoxin B	Bind MHC-II and TCRs, activates high levels of T cells leading to shock. Resists heat and acid	[8,17]
Alpha-hemolysin	Pore-forming cytolytic activity against erythrocytes, monocytes, macrophages, lymphocytes, fibroblasts, endothelial cells	[17]
Panton–Valentine leukocidin	Pore-forming cytolytic activity against polymorphonuclear cells, monocytes, macrophages	[17]
Exfoliatin	Cleave desmoglein1 to mediate staphylococcal skin disease	[17]
Clumping factors A and B	Play a critical role in attachment to valvular tissue and inducing platelet aggregation	[2]
Fibronectin-binding protein A and B	Facilitate binding to ECM fibronectin, facilitation endothelial inflammation and invasion and platelet aggregation	[2]
Serine aspartate repeat protein	Plays a role in platelet aggregation	[2]
Polysaccharide capsule	Present in 90% of clinical isolates, relevance as a virulence factor is debated	[17]

### 1.3. Vaccines

Vaccines for MRSA are an elusive but critical element in reducing the morbidity and mortality of the disease. Several vaccine candidates have ben reported in the literature, as summarized in Table 3 and discussed below. 

Reverse vaccinology approaches may significantly increase the number of potential vaccines and their efficacy. For example, Naorem et al. found two high-potential vaccine targets, PrsA and EssA, using reverse vaccinology approaches [19]. PrsA and EssA proteins are cell membrane proteins. PrsA is a foldase and is essential for MRSA cell wall protein folding; EssA is necessary for the secretion of EsxA protein and is likely to play a significant role in SA pathogenesis [19]. However, Miller et al. reported that studying and confirming specific antibody titers against superantigens and pore-forming toxins associated with negative outcomes is important to developing successful MRSA vaccines [5]. This illustrates that it is essential that advances in vaccine targeting driven by bioinformatics are balanced with real-world studies and real-world data.

Other promising vaccine targets include conjugated SA polysaccharides, SA iron-regulated surface determinant B, SA alpha-hemolysin, and Panton–Valentine leukocidin [20]. Lysins and leukocidins are exciting targets for toxoid vaccine approaches, especially as many opsonization-targeted vaccines have failed in clinical studies [5]. Tran et al. have reported that the multivalent toxoid vaccine (LukVax) produces high titers of neutralizing antibodies against alpha-hemolysin and Panton–Valentine leukocidin [21]. They also found that vaccination with LukVax protected against death in a rabbit model of MRSA necrotizing pneumonia [22]. Protection in mice against SA and MRSA infection and a robust Th1/Th17 immune response was also triggered by immunization with LukED and HlgAB toxoids [22].

Another study of a four-component vaccine targeting Panton–Valentine leucocidin, alpha-hemolysin, and recombinant toxic shock syndrome toxin in rhesus macaques found a robust IgG response, with the ability to neutralize Panton–Valentine leukocidin, alpha-hemolysin, toxic shock syndrome toxin, and other structurally similar SA antigens [23]. Additionally, the quadrivalent vaccine activated a balanced Th1/Th17 response through IL-17 and INF-gamma signaling, contributing to enhanced phagocytosis and bacterial clearance [23]. That study provides an interesting result because rhesus macaques are much more sensitive to staphylococcal toxins than mice [23]. Rhesus macaques are also colonized with SA and have a high frequency of antibodies already present to staphylococcal antigens [23]. The monkeys in that study showed very minimal adverse affect from vaccine administration and maintained good general health and normal leukocyte counts throughout the course of study [23]. Rhesus macaques also have similar major histocompatibility class II molecules, which increases the likelihood that similar effects will be present in humans [23]. This reason for this is twofold: First, the likelihood that human T and B cells will respond to the vaccine is increased [23]. Second, the MHC-II molecules binding staphylococcal superantigens is the primary mechanism for staphylococcal toxic shock, and the absence of shock symptoms in rhesus macaques makes the likelihood of shock syndrome upon vaccine administration less likely in humans [23].

Vaccine formulations, including Panton–Valentine leukocidin and alpha-hemolysin, are commonly studied; however, other conserved staphylococcal antigens may be critical to developing an effective vaccine. For example, Deng et al. studied a five-component vaccine named Sta-V5, containing the conserved antigens AdsA (recombinant), EsxA, EsxB, PmtA, and PmtC [24]. According to their study, the five-component vaccine produced a stronger immune response than any of the components individually. Additionally, mice immunized with Sta-V5 and subsequently subjected to lethal challenge with MRSA and SA intravenously had a more than 90% survival rate at the 30-day mark [24].

Another vaccine (4C-Staph) includes detoxified alpha-hemolysin, FhuD2, Csa1A, EsxA, and EsxB showed protection against SA challenge in a murine model [17]. In a murine model of neutropenia, 4C-Staph showed increased recruitment of macrophages and monocytes and effective compensation through the induction of a strong mononuclear response [17]. This shows promise for helping attenuate the effect of SA in patients with neutropenia.

Other toxoid vaccine approaches focus on toxins associated with the MRSA toxic shock syndrome (TSS). TSS toxin and staphylococcal enterotoxins B and C are strongly associated with MRSA toxic shock and fatality [16]. Vaccination of rabbits against staphylococcal enterotoxins B and C showed in vivo protection against lethal doses of wild-type staphylococcal enterotoxins [16]. Staphylococcal enterotoxins may be a critical component of future toxoid vaccines to protect against MRSA toxic shock syndrome. STEBVax is another staphylococcal enterotoxin B vaccine, using a recombinant toxoid that does not interact with human MHC receptors [17]. STEBVax protected mice against challenge with staphylococcal enterotoxin A, B, and C and SA toxic shock syndrome toxin [17]. Additionally, STEBVax showed strong immunogenicity and specific antibody response in phase I clinical trials.

SA iron-regulated surface determinant B is a highly conserved surface protein [17]. V710 is a vaccine developed targeting SA iron determinant B studied in cardiothoracic surgery patients in phase II and III trials [17]. Despite being highly immunogenic in animals, V710 increased mortality rates in study patients [17]. This is likely due to decreased IL-2 and IL-17 and diminished Th17 responses [17]. Future vaccines targeted toward SA iron surface determinant should be evaluated for effects on IL-2, IL-17, and Th-17 response before advancing to clinical trials.

Another target, a staphylococcal lysin, PBP2a, plays a critical role in the hydrolysis of peptidoglycan during the fission of bacterial daughter cells [25]. Recombinant PBP2a autolysin conjugated with PGLA nanoparticles produced high titers of neutralizing antibodies in a murine model. Additionally, high levels of opsonophagocytosis and increased survival were reported for mice challenged with SA intraperitoneally [25].

MRSA and SA infections can present either systemically or locally and may present in a variety of tissues; vaccines aimed toward preventing certain types of infections in certain groups may be important. For example, mice immunized via an intrapulmonary route with staphylococcal clumping factor A showed increased IL-17 and long-lasting Th17 responses in the lung parenchyma [26]. Fan et al. also found a 50% survival rate for pulmonary SA challenge among mice immunized with an intrapulmonary vaccination compared with a 10% survival rate at 3 days among intranasally immunized mice [26].

Vaccine conjugates are also critical in driving sufficient immune response against SA. Fungal beta-glucan is a potentially helpful adjuvant, as it is active as a fungal pathogen-associated molecular pattern [27]. Mice immunized with ClfA, IsdA, MntC, and SdrE antigens, all linked to beta-glucan molecules, efficiently produced proinflammatory cytokines and long-lasting (at least 8 weeks) cellular and humoral immune responses [27]. Additionally, mice injected with rMntC-EPS30, a recombinant MtnC vaccine combined with *Lactobacillus casei* exopolysaccharides as an adjuvant, provided a robust immune response [28]. In mice treated with rMntC-EPS30, first by the subcutaneous route, followed by the intranasal route, about 90% survived upon an SA pulmonary challenge [28].

SA polysaccharide capsules may be another important target for the development of an SA vaccine. SA capsular polysaccharides CP5 and CP8 with detoxified *Pseudomonas aeruginosa* exotoxin A (StaphVAX) were studied in a phase III trial with patients undergoing cardiothoracic surgery [17]. StaphVAX was administered at different intervals before cardiothoracic surgery and reduced SA bacteremia by 64% at 32 weeks, by 57% at 40 weeks, and by 26% at 54 weeks [17]. These findings suggest the potential for future success with SA polysaccharide conjugate vaccines.

Other vaccine candidates also contain capsular polysaccharides in their formulations. Pfizer developed the SA4Ag vaccines using adhesion molecule ClfA, manganese transporter MntC, and CP5 and CP8 [17]. Phase I and II clinical trials indicated the safety and immunogenicity of SA4AG, including initial evidence of efficacy in patients with spinal fusion [17]. Additionally, SA4AG was found to provide a functional immune response for up to 3 years in healthy adults [17]. Despite these positive findings, further trials with this vaccine did not demonstrate efficacy in preventing surgical SA infection [17].

Another polysaccharide conjugate vaccine consisting of CP5 and CP8 conjugated to an alpha-hemolysin–manganese transporter c fusion protein (HLA-MntC-SACOL0723) produced a strong IgG response in a murine model [29]. Additionally, HLA-MntC-SACOL0723 showed improved opsonophagocytosis compared with polysaccharide immunization alone and a reduced bacterial load following SA challenge [29]. Another vaccine study consisted of CP5 and CP8 conjugated to a non-toxic mutant of diphtheria toxin (CRM) [30]. In a murine model testing CP5-CRM and CP8-CRM, vaccination with either of the CP5-CRM and CP8-CRM components protected against SA bacteremia; however, neither CP5-CRM nor CP8-CRM protected against lethal sepsis [30].

A phase 1 randomized controlled trial investigated a multicomponent polysaccharide conjugate vaccine developed by GSK vaccines containing CP5, CP8, tetanus toxoid, alpha-hemolysin toxoid, and ClfA with ASO3B adjuvant [31]. The randomized controlled trial showed no safety concerns and demonstrated robust antibody responses to each staphylococcal antigen after one dose and a stable antibody concentration through to the study’s end [31]. Unfortunately, the trial results showed low levels of IL-17 activity in response to vaccination, suggesting that protection against severe SA disease may be limited [31]. Further clinical studies will need to be performed to assess the efficacy of GSK’s SA vaccine.

**Table 3:** Vaccine candidates against SA and MRSA infections.

Vaccine Name	Type	Antigens	Reference
LukVax	Toxoid	Alpha-HL, PVL	[21]
N/A	Toxoid	LukED, HlgAB	[22]
IBT-V02	Toxoid	Alpha-HL, PVL, TSST	[23]
STA-V5	Conserved antigens	AdsA, EsxA, EsxB, PmtA, PmtC	[24]
4C-Staph	Combination	Alpha-HL, FhuD2, Csa1A, EsxA, EsxB	[17]
N/A	Toxoid	SEB	[16]
STEBVax	Toxoid	Recombinant SEB	[17]
V710	Conserved antigen	Iron surface determinant B	[17]
N/A	Conserved protein	PBP2a autolysin	[25]
N/A	Intrapulmonary staphylococcal clumping factor A	Staphylococcal clumping factor A	[26]
N/A	Conserved antigen- conjugate	ClfA, IsdA, MntC, SdrE, beta glucan conjugate	[27]
rMntC-EPS30	Conserved antigen conjugate	MntC–*Lactobacillus casei* exopolysaccharide conjugate	[28]
StaphVAX	Capsular polysaccharide conjugate	CP5, CP8, and P. aeruginosa exotoxin A conjugate	[17]
SA4Ag	Conserved protein and capsular polysacharides	ClfA, MntC, CP5, CP8	[17]
HLA-MntC-SACOL0723	Toxoid, conserved protein, and capsular polysacharides	HLA–MntC fusion protein, CP5, CP8	[29]
N/A	Conjugate polysacharide	CP5, CP8, diphtheria toxoid conjugate	[30]
GSK2392103	Toxoid, conserved protein, capsular polysaccharides, and adjuvant	Tetanus toxoid, mutant alpha-hemolysin, ClfA, CP5, CP8	[31]

N/A = not available.

### 1.4. Immunotherapies

Immunotherapies include many forms of treatments aimed at modulating or affecting normal immune processes. Vaccines are the most common and oldest form of immunotherapy [32]. Many targets for vaccination derived from animal models have failed to produce an effective vaccine against SA and MRSA infections in human studies [33]. However, many of these vaccine targets may be effective monoclonal antibody targets due to the lack of a need for the host immune system to develop effective antibodies against SA. Additionally, as our understanding of immune changes involved in MRSA pathogenesis improves, treatments aimed at reversing the MRSA immune evasion process may significantly improve outcomes. 

In the absence of an effective and FDA-approved vaccine, other immunotherapies are crucial for reducing the morbidities and mortalities due to MRSA infections. Some examples of newer forms of immunotherapies include monoclonal antibodies, checkpoint blockade therapy, cytokine therapy, and chimeric antigen receptor T cells (Table 4) [32]. Monoclonal antibodies may be used either to facilitate opsonophagocytosis and neutralize microbial toxins or in checkpoint blockade such as anti-PD-1/PDL-1 monoclonal antibodies [32]. Cytokine therapy involves the administration of pro-inflammatory cytokines to address deficits in the cytokine response [32]. Chimeric antigen receptor T cells are produced in vitro and introduced in vivo to induce cell-mediated responses against specific targets [32].

As potent SA toxins, alpha-hemolysin and Panton–Valentine leukocidin are critical targets for monoclonal antibody approaches and vaccine approaches. A study of three novel Panton–Valentine leukocidin and two novel alpha-hemolysin monoclonal antibodies found high synergy and protective effects in a rodent model of SA pneumonia [34]. Staphylococcal enterotoxin B is also a critical neutralization target for monoclonal antibody approaches, as it produces high levels of proinflammatory cytokines and is involved in food poisoning, toxic shock syndrome, and immune evasion [35]. In a murine toxic shock model, 100% of mice injected with anti-staphylococcal enterotoxin B monoclonal antibody (M0313) survived, while over 90% of control mice died [35]. IL-2, IL-6, IFN-γ, and MCP-1 expression was also significantly lower in mice given M0313 [35].

The successful targeting of multiple staphylococcal superantigens is important for monoclonal antibody approaches. De-speciated IgG produced from rabbits immunized with the IBT-V02 (IBT-V02-F(ab’)2) showed the ability to neutralize the leukotoxins HlaAB, HlaCD, and LukED and the superantigens SEC1, SED, SEK, and SEQ [36]. Mice treated with IBT-V02-F(ab’)2 in a model of MRSA bacteremia and sepsis had significantly higher survival rates than controls [36].

SA structural molecules are also essential markers and targets for immunotherapeutics. Lipoteichoic acids are found on the cell membranes of Gram-positive bacteria. ZBIA3H is a lipoteichoic acid-binding monoclonal antibody, and, in combination with the antimicrobial linezolid, it produced a 63% survival rate against lethal challenge in an MRSA sepsis mouse model and a higher overall survival rate than linezolid or ZBIA3H alone [37]. Anti-lipoteichoic acid monoclonal antibodies have failed in previous clinical trials, suggesting that combination therapies may enhance the efficacy of monoclonal antibodies [37].

MRSA or SA pneumonia secondary to influenza produces a synergistic inflammation and injury to the lung, producing damage greater than either influenza or SA produce on their own [38]. SA alpha-hemolysin may also be critical to the pathogenesis of secondary SA pneumonia, as treatment with an alpha-hemolysin-targeting monoclonal antibody (MEDI4893) resulted in protection against secondary MRSA pneumonia in a mouse model [38].

Radioimmunotherapy is a potentially promising new approach for treating SA and MRSA infections. For example, the non-specific lipoteichoic acid monoclonal antibody palivizumab paired with bismuth-213 showed significant clearance of MRSA planktonic cells and biofilm [39]. The ability of MRSA to form a biofilm is a potentially devastating complication in joint replacements, and the use of radioimmunotherapy may significantly improve treatment for these types of infections if validated by clinical studies [39]. Radioimmunotherapy through radioactive monoclonal antibodies may also be a promising area of study for other types of MRSA infections.

Other approaches, such as anti-staphylococcal lysins, are also essential tools for treating MRSA infections and may be a critical component of future combination therapy protocols [40,41]. Exebacase (Lysin CF-301), an anti-staphylococcal lysin, eradicates biofilms and has synergistic effects with antibiotics [42]. The use of exebacase in a human randomized controlled trial demonstrated protection against MRSA [40]. Patients with MRSA given exebacase combined with standard-of-care antibiotics had a 3.7% versus a 25% all-cause mortality compared to patients who only received standard-of-care antibiotics [40]. Less significant differences were found in patients with methicillin-sensitive SA, suggesting that differences may occur in efficacy between methicillin-sensitive SA and MRSA [40]. These differences also prove a need for additional clinical studies to confirm the effectiveness of exebacase.

Immune over-response is also an essential part of the damage MRSA and SA cause to those infected, and this presents an opportunity for novel immunotherapeutics, such as enzyme inhibitors aimed at attenuating harmful immune responses. Caspases are critical to triggering proinflammatory responses and may contribute to significant host damage due to inflammation in SA [43]. The pancaspase inhibitor Q-VD-OPH inhibits several caspases involved in the MRSA immune response. In a mouse model of MRSA, skin infections showed decreased skin lesion sizes and decreased necrosis [43]. Q-VD-OPH also increased macrophage recruitment through an interleukin-1B-independent mechanism, suggesting that the host immune response mediated by caspases in the skin may detrimentally inhibit macrophage activity [43].

Targeted gene therapy to knock out harmful cytokines is another promising future treatment for SA and MRSA infections. Silicon nanoparticles loaded with short interfering RNAs are a potentially potent modulator of immune responses [44]. A gene therapy approach using macrophage-targeted silicon nanoparticles loaded with anti-Irf5 gene was found to improve the outcome of SA infections in mice significantly [44]. The depletion of Irf5 significantly decreased inflammation and prevented an excessive immune response in treated mice [44]. This allowed mice treated with Irf5 siRNA to clear abscesses in a musculoskeletal infection challenge [44]. It is suspected that decreased Irf5 leads to decreased macrophage over-activation and enhances the ability of the immune system to clear infections and abscesses [44]. Specific pitfalls to gene therapy in humans with MRSA infection include a lack of human data, the possibility of different responses in the human host, and differential penetration of the therapy in a large organism. Human studies may also be difficult to obtain approval for due to the novelty of the treatment approach, and they may be confounded by the coadministration of antibiotics, which is the standard of care.

**Table 4:** Immunotherapies against SA and MRSA infections.

Name of Immunotherapy	Type	Targets	References
N/A	Antitoxin mAb	Alpha-HL, PVL	[34]
N/A	Antitoxin mAb	SEB	[35]
IBT-V02	Mixed de-speciated IgG	Multiple	[36]
ZBIA3H	Anti-cell-surface antigen mAb	LTA	[37]
MEDI-4893	Antitoxin	Alpha-HL	[38]
N/A	Radioactive mAb (bismuth-213)	LTA	[39]
Exebacase	Lysin	N/A	[40,42]
Q-VD-OPH	Pancaspace inhibitor	Multiple caspaces	[43]
N/A	Si-RNA	Irf5	[44]

N/A = not available. Note: All except for exebacase are currently in the preclinical phase of testing.

## 2. Discussion

MRSA is a highly complex organism, and its mechanism of pathogenesis is still not fully understood. The diversity of different antibiotic resistance and virulence factors that a particular strain may carry, in addition to a jungle of different data from animal models, makes developing a clear and concise treatment strategy complicated. The ability of MRSA to rapidly evolve antibiotic resistance may also result in the development of resistance to vaccines or new therapies such as lysins or immunotherapeutics. The same genetic mechanisms of transduction, conjugation, and transformation may result in resistance to vaccines and immunotherapies in environments in which these treatments may be used due to the same microevolutionary forces that caused the dissemination of the original MecA gene. Additionally, due to a heavy reliance on animal data and in vitro studies, it is difficult to predict the efficacy of novel MRSA vaccine and immunotherapy candidates.

In the future, toxoid approaches to MRSA may be a promising area of further research for three reasons. Firstly, toxoids are highly immunogenic; multiple studies included in this review showed impressive toxin neutralization [16,21,23]. Second, toxoid approaches may not be as significantly affected by MRSA immune evasion strategies such as SA protein A. Finally, a study of rhesus macaques showed that a toxoid vaccine may allow modulation of the MRSA immune response by allowing a robust IFN-gamma, IL-6, and IL-17 response [23]. A robust IFN-gamma, IL-6, and IL-17 response may allow for a strong cell-mediated immune response, which is likely to be more effective against MRSA, given the ability of MRSA protein A to defend against humoral immunity.

The limited clinical trial success for MRSA vaccines may also be due to the need for better adjuvants used in vaccine formulations. Dendritic cells exposed to heat shock inactivated SA produced a response 6 to 10 times less than those exposed to live SA [13]. The potential for increasing and/or modulating the immune response to MRSA vaccine candidates through novel adjuvants could improve real-world efficacy. Two studies included in this review took this approach and found robust immune responses to fungal beta-glucan and *Lactobacillus casei* polysaccharide adjuvants [27,28]. Fungal beta-glucan combined with SA ClfA, IsdA, MntC, and SdrE produced a strong Th1-Th17 immune response, which protected against a systemic SA challenge in mice [27]. This is suspected to be due to the activation of innate pattern recognition receptors against the beta-glucan pathogen-associated molecular pattern (PAMP) [27]. Additionally, *Lactobacillus casei* exopolysaccharide combined with MntC produced a strong Th1-Th17 cytokine response and protection against SA in pulmonary and cutaneous infection challenges in mice; this is likely due to the recognition of *Lactobacillus casei* exopolysaccharide as a PAMP [28].

Additionally, several vaccines discussed in this review included capsular polysaccharide antigens in their formulations. Capsules are present in roughly 90% of SA clinical isolates and may be a useful target for vaccines [17]. Multiple candidates containing CP5 and CP8 (StaphVAX, SA4Ag) showed promising but inconclusive results in clinical trials [17]. Additionally, polysaccharide capsule vaccines may not provide coverage for USA300 MRSA due to the absence of a capsule in this strain [18]. 

However, while a successful vaccine for MRSA would be the gold standard for preventing MRSA mortality, innovation in other areas will likely continue to be necessary even if such a vaccine is successful in future trials. Without a proven vaccine, other strategies become more critical to developing new non-antibiotic treatments. Antitoxin monoclonal antibodies are a critical piece of novel approaches for the treatment of MRSA infections. The appeal of these treatments is the same as the appeal for toxoid vaccines. Several antitoxin monoclonal antibodies included in this review demonstrated high efficacy in murine models of MRSA infection [34,35,36,38]. Clinical studies will be necessary to evaluate whether monoclonal antibody therapy is useful in MRSA. The limitations of this approach include the specificity of monoclonal antibodies to specific antigens, which may be ineffective in combating a pathogen with the ability to produce many different toxins. Additionally, achieving and maintaining effective inhibitory concentrations of monoclonal antibodies may be difficult in a larger host with a higher volume of distribution such as a human.

Other innovative immunotherapies may lead to improved outcomes as well. We understand that specific immune responses, such as Th1/Th17 balance, and appropriate proinflammatory cytokine response correlate with favorable outcomes in MRSA and SA infections [45]. Modulators of the host immune response that lead to these responses and prevent damage through the over-expression of proinflammatory cytokines may make a significant difference. Two studies in this review discussed enzyme inhibition and gene therapy that showed successful results in animal models [43,44]. However, further studies of these immune-modulating therapies, especially clinical studies, are necessary to confirm these findings.

## 3. Conclusions

The current literature for the understanding of immune responses and the development of vaccines, and immunotherapies for SA and MRSA infections relies heavily on animal models. However, the literature shows promise for toxoid vaccine approaches, antitoxin monoclonal antibodies, and novel therapies to modulate the host immune response to MRSA. Toxoid vaccines may provide protection against toxic shock and other clinical manifestations of systemic MRSA infections, allowing the patient’s own immune system to clear the infection. Toxoid vaccine may also prime the immune system to respond appropriately to SA infections, potentially reducing the risk of severe outcomes if the same results are found in human studies. Monoclonal antitoxin antibodies may also provide protection against severe outcomes due to SA toxin production if the same results are found in human studies. Challenges in bringing these therapeutics from mouse models to the bedside include differences in human and murine physiology and immunology, differences in the size of humans and mice, comorbidities present in human patients, and challenges associated with trialing new treatments in critically ill patients. The literature also indicates that a balanced Th1/Th17 response and an appropriate expression of proinflammatory cytokines is predictive of positive outcomes in SA and MRSA infections. With regard to the future, developing multivalent toxoid vaccines, antitoxin monoclonal antibody products, and immune-modulating therapies are promising research areas and have potentials for improving outcomes for patients infected with SA and MRSA.
